# Incidence of hepatitis C virus infection and associated determinants among men who have sex with men without HIV in Amsterdam, the Netherlands, between 2012 and 2021

**DOI:** 10.1097/MEG.0000000000003008

**Published:** 2025-06-23

**Authors:** Kris Hage, Jeffrey Koole, Anders Boyd, Amy Matser, Udi Davidovich, Margreet Bakker, Lia van der Hoek, Jelle Koopsen, Sjoerd Rebers, Janke Schinkel, Maria Prins

**Affiliations:** a Department of Infectious Diseases, Public Health Service of Amsterdam; b Department of Infectious Diseases, Amsterdam UMC, University of Amsterdam; c Amsterdam Institute for Immunology and Infectious Diseases; d Stichting hiv monitoring; e Department of Social Psychology, University of Amsterdam; f Department of Medical Microbiology and Infection Prevention, Amsterdam UMC, University of Amsterdam, Amsterdam, The Netherlands

**Keywords:** HCV, MSM without HIV, phylogenetic analysis

## Abstract

**Objective:**

To assess the hepatitis C virus (HCV) prevalence, incidence, and associated determinants among men who have sex with men (MSM) without HIV in Amsterdam, the Netherlands.

**Methods:**

We used data from the Amsterdam Cohort Studies (2012–2021) to calculate the prevalence of past/current HCV infection at the first study visit and incidence rate of primary HCV infection during follow-up. We identified determinants associated with incident HCV infection using univariable Bayesian exponential survival models. Phylogenetic analysis was conducted to compare HCV sequences of MSM without HIV to those from MSM with HIV and those using HIV pre-exposure prophylaxis.

**Results:**

A total of 926 MSM were included. At first visit, 2/926 (0.2%) had a past/current HCV infection. Among 891 participants contributing to 6083.30 person-years of follow-up, three incident HCV infections were observed (incidence rate = 0.05/100 person-years). These infections were observed between 2014 and 2018, and all participants had never used HIV pre-exposure prophylaxis. Incident infections were associated with receptive condomless anal sex, having 1–10 sexual partners vs. none, recent injecting drug use (IDU), ever IDU, and fisting, albeit there was substantial uncertainty for all determinants (i.e. 95% credible intervals included one). Phylogenetic analysis revealed that one HCV-RNA sequence was closely related to HCV sequences from MSM with HIV.

**Conclusion:**

While HCV infection is uncommon among MSM without HIV, the risk of infection seems to increase among those with specific behaviors. HCV screening for MSM without HIV should be focused on those reporting these behaviors.

## Introduction

In high-income countries, such as the Netherlands, men who have sex with men (MSM) are disproportionally affected by HIV and other sexually transmitted infections (STIs), including hepatitis C virus (HCV) [1]. HCV, a blood-borne infection (BBI), can cause severe liver disease when left untreated. Since 2000, HCV has emerged as an STI among MSM, particularly among those with HIV [2,3]. Previously, HCV was treated with a 48-week course of pegylated interferon (peg-IFN) [4]; however, peg-IFN treatment caused severe side-effects and was, depending on HCV genotype, often not successful [5]. Treatment with direct-acting antivirals (DAAs), leading to higher rates of sustained virologic response and with much lower toxicity compared with peg-IFN, became clinically available in 2015 and its widespread treatment uptake substantially reduced new HCV infections [6,7].

Globally, an estimated 6% of MSM with HIV have tested anti-HCV antibody positive [8]. HCV transmission in this group is largely through sexual contact, with behaviors such as receptive condomless anal sex (CAS), a higher number of lifetime sex partners, recent STI, engaging in sexual behaviors associated with damage of the anorectal mucosa (e.g. use of anal sex toys) and sexualized drug use (SDU) being associated with HCV acquisition [9–11]. Until recently, HCV had been infrequently observed in those without HIV [12]; however, the development and implementation of new HIV biomedical prevention strategies in the last decade, including treatment as prevention and HIV pre-exposure prophylaxis (PrEP), has resulted in more frequent overlap in sexual networks between MSM with and without HIV [13–15] and an increase in HCV acquisition among MSM without HIV, as evidenced by the comparably high prevalence and incidence rate of HCV among MSM using PrEP to MSM with HIV [12].

Between 1984 and 2012, a previous study among MSM without HIV in Amsterdam, the Netherlands, observed no HCV infections [16]. One study conducted in Amsterdam among MSM without HIV using PrEP between 2015 and 2018 reported an HCV incidence of 2.3 per 100 person-years [17]. It is unclear whether HCV infections are also circulating in MSM with arguably lower risk of acquiring HCV infection. A Dutch study among 1885 MSM without HIV and not using PrEP attending four different Centers of Sexual Health outside the Amsterdam region between 2019 and 2022 observed an anti-HCV antibody prevalence of 0.05% [18]. However, sexual contacts and risk behaviors among MSM without HIV in Amsterdam, with its higher prevalence of MSM and MSM meeting places, could occur more frequently and the risk of external introductions of HCV is higher [19]. This study then aimed to estimate the prevalence and incidence of primary HCV infections, along with their associated determinants, among MSM without HIV participating in the Amsterdam Cohort Studies (ACS) between 2012 and 2021. Furthermore, we intended to evaluate whether strains of HCV infections observed in this study clustered with strains circulating among MSM with HIV and MSM without HIV using HIV PrEP.

## Methods

### Study population and design

The ACS is an ongoing open, observational, prospective cohort study among MSM with and without HIV in/around Amsterdam, starting in 1984. It aims to investigate the epidemiology of HIV and other STIs and BBIs, and to evaluate the effect of interventions to reduce its transmission. Detailed information about the study procedures has been described elsewhere [20,21]. Briefly, MSM aged 16 years or older living in or regularly participating in MSM-related activities in the Amsterdam area are eligible for study participation. Recruitment is based on convenience and chain referral sampling. Study participation is voluntary and without incentive. Written informed consent is obtained at study enrollment. The ACS has been approved by the Medical Ethics Committee of the Amsterdam University Medical Center of the University of Amsterdam, the Netherlands (MEC 07/182).

For this study, we included all MSM without HIV using PrEP or not participating in the ACS between 1 January 2012 and 31 December 2021.

### Data collection

Prior to study visits, participants were requested to complete a self-administered questionnaire on sexual health and behavior, including recent SDU and PrEP use. During these visits, blood samples were drawn for HIV and STI testing and stored at −80 °C. Urinal, rectal, and pharyngeal samples were self-collected for STI testing. Detailed sampling and laboratory testing procedures have been described elsewhere [21]. Routine HCV testing among MSM without HIV in the Netherlands is limited to those using PrEP, those with a lymphogranuloma venereum diagnosis, or in case of a partner notification [22,23]. Between study visits, participants could visit the Centre for Sexual Health at the Public Health Service of Amsterdam for additional STI and HCV testing if there was an indication for testing. Data collected at both the ACS study visits and additional STI visits were used for this study.

We used a back-testing algorithm to assess HCV infection and minimize the width of the seroconversion interval. Because only blood samples collected during ACS study visits were stored for retrospective analysis, only these samples were available for HCV back-testing. Anti-HCV antibody testing was performed using Liaison XL MUREX HCV Ab (DiaSorin, Saluggia, Italy) and HCV-RNA using a home-based PCR. For those participants without available anti-HCV antibody results during follow-up, we tested the most recent stored blood sample for anti-HCV antibodies. If positive, additional samples were then sequentially back-tested to identify the most recent anti-HCV antibody negative sample. Finally, we took the first anti-HCV antibody positive sample and tested it for the presence of HCV-RNA. Samples with a borderline positive anti-HCV antibody signal at baseline (for those with only one ACS study visit) or consecutive ACS study visits (for those with ≥2 ACS study visits during follow-up) were considered anti-HCV antibody negative in our analysis. This classification was based on the interpretation that persistent borderline positive anti-HCV antibody signals in patients who were neither diagnosed with nor treated for an HCV infection likely represent nonspecific signals [24]. On finding new prevalent or incident HCV infection using retrospective testing, HCV awareness and HCV treatment history was checked with participants.

### Hepatitis C virus genotyping and phylogenetic analysis

We isolated HCV-RNA from 140 µl of plasma using the Viral RNA Mini Kit (Qiagen, Germantown, Maryland, USA), with elution in 60 µl. The isolated RNA was reverse transcribed into cDNA using Viloscript (Invitrogen), which was then genotyped with DM100 and DM101 primers [25]. For full-length sequencing, six primer pools were used to amplify 4 µl of cDNA per pool at 64 °C for 40 cycles (Q5 High-Fidelity DNA Polymerase; New England Biolabs). These pools were then combined. A total of 150 ng per sample was sequenced on a Nanopore R9 flowcell in combination with the Rapid Barcoding Kit (SQK-RBK-111.96). Phylogenetic trees were constructed by comparing the HCV sequence obtained to a global set of recent HCV infections [26]. In addition, we compared the HVR1 fragment of the HCV sequence to HVR1 sequences obtained from MSM using PrEP in the Amsterdam PrEP project (2015–2018) [13,17]. Maximum-likelihood phylogenies were constructed using a general time-reversed substitution model with γ-distribution, assuming a certain fraction of evolutionary invariable sites (GTR+G+I) in IQTREE v2.3.6. Ultrafast bootstrapping (*n* = 1000) was performed to analyze the stability of tree topology. Trees were visualized in R and the ggtree package [27].

### Study covariables

We collected data on age (in years), country of birth (Netherlands vs. other), PrEP use (yes/no), educational level (below college degree vs. college degree or higher), receptive CAS (yes/no), number of sexual partners, recent IDU (yes/no), ever IDU (yes/no), chemsex (yes/no), any SDU (yes/no), chlamydia, gonorrhea or syphilis diagnosis (i.e. ‘any STI’, yes/no), fisting (yes/no), use of anal sex toys (yes/no), and sharing of anal sex toys (yes/no). Chemsex was defined as self-reported sexualized use of methamphetamine, γ-hydroxybutyric acid/γ-butyrolactone, mephedrone, ketamine, amphetamine, or ecstasy. Any SDU excluded alcohol, nitrates (poppers), erection stimulants, and cannabis use. All covariables, except country of birth, educational level, and ever IDU were time-updated. Behavioral factors referred to the 6 months preceding the study visit.

### Statistical analysis

Using data from MSM with at least one ACS study visit after 1 January 2012, we defined ‘baseline’ as the first ACS study visit since January 2012. We excluded those who identified as transgender. Prevalent HCV infection was defined as having an anti-HCV antibody or HCV-RNA positive sample (i.e. past/current HCV infection) at baseline. To calculate the prevalence of HCV infection at baseline, the number of individuals with past/current HCV infection was divided by the total number of individuals with a baseline visit. We used the binomial exact method to calculate 95% confidence interval (CI) for the prevalence.

Using data from MSM with at least two ACS study visits, follow-up began at baseline and continued until the estimated date of HCV infection, date of HIV infection for those who seroconverted HIV-positive during follow-up, or the most recent study visit, whichever occurred first. Participants with past/current HCV infection at baseline were excluded. Incident HCV infection was defined as having an anti-HCV antibody or HCV-RNA positive result preceded by an anti-HCV antibody negative result. Only the first incident HCV infection was included in the analysis (i.e. primary HCV infection). The date of incident primary HCV infection was estimated using the midpoint between the last negative and first positive test. To calculate the incidence rate of primary HCV infection, the number of incident infections was divided by the total person-years of follow-up and expressed per 100 person-years. The exact Poisson 95% CI was calculated for the incidence rate.

In the deterministic analysis, we anticipated few incident HCV infections and thus, parameter estimates from standard regression techniques would be biased away from actual estimates (i.e. ‘sparse data bias’ [28]). To minimize this bias, a penalized regression approach was used whereby uncertain estimates from the data are pulled toward more realistic ones assumed from prior knowledge [29]. Briefly, univariable Bayesian exponential survival models were fit for each covariate separately, and priors for hazard ratio (HR) distributions were based on their anticipated strength of association (Supplementary Table S1, Supplemental digital content 1, https://links.lww.com/EJGH/B175) [29,30]. These distributions were assigned to covariates based on previous studies conducted among MSM with HIV and MSM using PrEP in the Netherlands [9,17,31]. Where there was no available data, we assumed a prior distribution with no association (Supplementary Table S1, Supplemental digital content 1, https://links.lww.com/EJGH/B175). Because of limited data, the prior for the distribution of the model intercept (i.e. the incidence rate for HCV infection without covariates) was uniformly flat. Using these priors and the data, a posterior median HR was estimated along with 95% credible intervals (CrI) using Markov Chain Monte Carlo methods. This model was estimated using the ‘bayes’ prefix command in Stata. Missing data on covariates were imputed by carrying forward responses from the preceding study visit with available data (i.e. last observation carried forward). If data remained missing, we assumed the missing value represented either the absence of the covariate (dichotomous variables) or the most frequently occurring category (categorical variables). See Supplementary Table S2, Supplemental digital content 1, https://links.lww.com/EJGH/B175 for the number of missing values for each imputed covariate.

All statistical analyses were performed using Stata (version 17.0, College Station, Texas, USA).

## Results

### Description of the study population

**Fig. 1.**
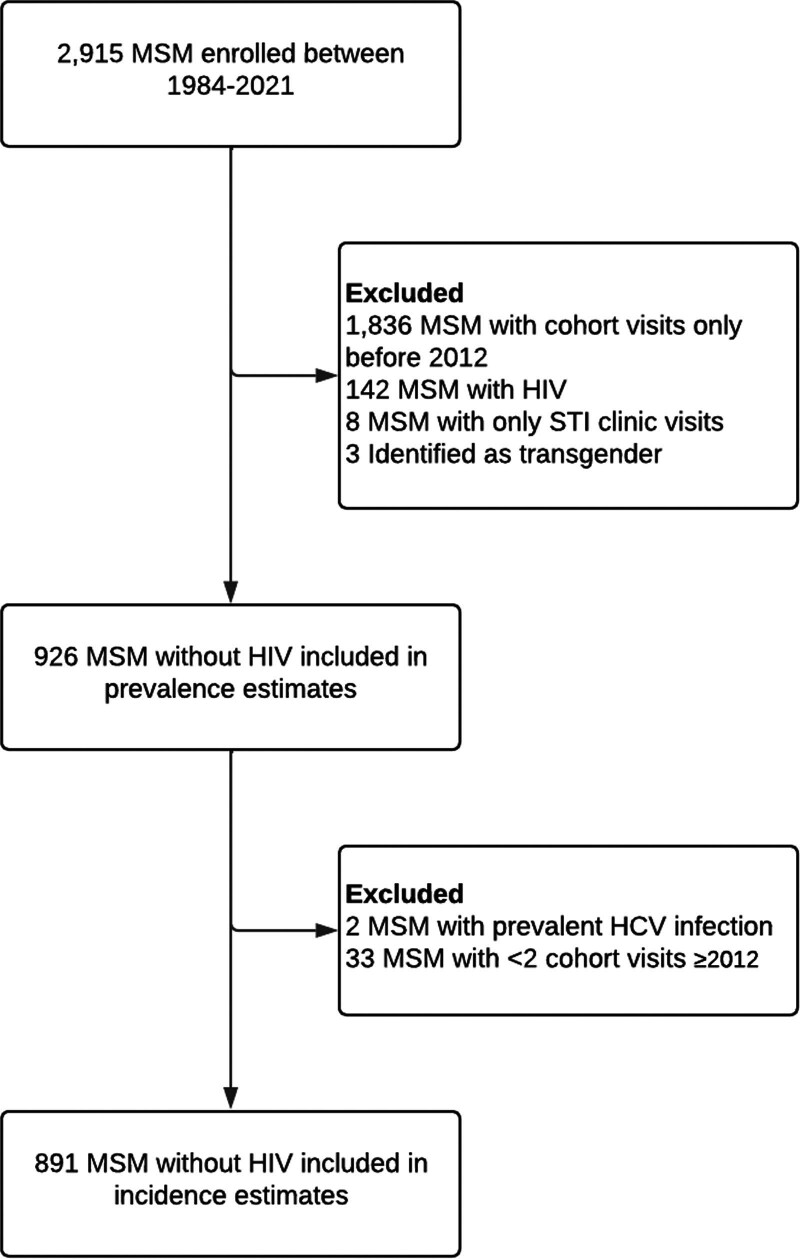
Flowchart of study participants. HCV, hepatitis C virus; MSM, men who have sex with men; STI, sexually transmitted infection.

Of the 2915 MSM who were ever included in the ACS, 926 were HIV-negative and had at least one ACS study visit between 1 January 2012 and 31 December 2021 (Fig. 1). Characteristics of the study population at baseline are shown in Table 1. Median age at baseline was 36 years [interquartile range (IQR) = 28–43]. The majority of participants (*n* = 765, 82.6%) were born in the Netherlands and had obtained a college/university degree or higher (*n* = 730, 78.8%). At baseline, 98.4% had never used PrEP (i.e. PrEP naive).

**Table 1.** Characteristics of 926 men who have sex with men without HIV participating in the Amsterdam Cohort Studies at baseline, Amsterdam, the Netherlands, 2012–2021

	Total (*n* = 926)
Year of baseline visit	2012 (2012–2015)
Age (years)^a^	36 (28–43)
18–34	399 (43.1)
35–44	333 (36.0)
≥45	183 (19.8)
Born in the Netherlands^a^	765 (82.6)
Highest educational level^a^	
No college degree	194 (21.0)
College/university degree	730 (78.8)
Residence in Amsterdam^a^	752 (81.2)
Living situation^a^	
Alone	454 (49.0)
With steady partner	293 (31.6)
With parents or caretakers	31 (3.4)
With others	144 (15.6)
Exclusively homosexual^a^	736 (79.5)
PrEP naive	911 (98.4)

Presented are *n* (%) or median (IQR).

IQR, interquartile range; PrEP, pre-exposure prophylaxis.

a Missing data: age, *n* = 11; country of birth, *n* = 9; educational level, *n* = 2; residency, *n* = 12; living situation, *n* = 4; sexual orientation, *n* = 5.

### Prevalent hepatitis C virus infection at baseline

Of the 926 participants with at least one visit between 2012 and 2021, two (0.2%) had a past/current HCV infection at baseline (95% CI = 0.03–0.78%). Both were anti-HCV antibody positive with a negative HCV-RNA result, suggesting past infections. Both participants were PrEP naive at baseline (Table 2). Three participants had borderline positive anti-HCV antibody signals at baseline (*n* = 1 with one study visit) or at baseline and consecutive visits (*n* = 2 with ≥2 study visits during follow-up) and were not considered as prevalent HCV infection (range signal-to-cutoff between 1.00 and 5.40).

**Table 2.** Characteristics of men who have sex with men without HIV who had a past/current hepatitis C virus infection at baseline or acquired an hepatitis C virus infection during follow-up in the Amsterdam Cohort Studies, 2012−2021

	Past/current HCV infection at baseline		Incident HCV infection during follow-up		
	Participants		Participants		
	1	2	3	4	5
Year of ACS enrollment	2008	2012	2014	2005	2013
Year of baseline visit	2012	2012	2014	2012	2013
Year of incident HCV infection	NA	NA	2018	2017	2014
Presence of HCV-RNA	No	No	No	Yes	Yes
PrEP naive at moment of HCV diagnosis	Yes	Yes	Yes	Yes	Yes
Ever indicated the use of PrEP after HCV seroconversion	No	No	No	No	Yes
Year of start using PrEP	NA	NA	NA	NA	2018
Initial PrEP regimen	NA	NA	NA	NA	Event-driven
Acquired HIV after HCV seroconversion	No	No	No	No	No

ACS, Amsterdam cohort studies; HCV, hepatitis C virus; NA, not applicable; PrEP, pre-exposure prophylaxis.

### Incident primary hepatitis C virus infection during follow-up

Of the 926 participants with a study visit between 2012 and 2021, 35 were excluded from the analysis on incidence of HCV as they did not have at least two study visits during follow-up (*n* = 33) or they had past/current HCV infection at baseline (*n* = 2). The 891 MSM included in the HCV incidence analysis were older (*P* = 0.04) and more often lived with a steady partner versus living alone (*P* = 0.02), compared to those excluded (Supplementary Table S3, Supplemental digital content 1, https://links.lww.com/EJGH/B175).

Median follow-up time was 7.7 years (IQR = 4.6–9.3 years), totaling 6083.30 person-years at risk for HCV infection. Of 891 MSM at risk, 21 MSM acquired HIV during follow-up. There were three incident primary HCV infections (overall incidence rate = 0.05/100 person-years, 95% CI = 0.02–0.15). Of the three incident HCV infections, two were both anti-HCV antibody and HCV-RNA positive, suggesting active infections, and one was anti-HCV antibody positive and HCV-RNA negative, suggesting spontaneous clearance (Table 2). No information on HCV treatment was available for the two participants with active infections. Median age at the estimated date of incident HCV infection was 44 years (IQR = 41–61 years). All three participants were PrEP naive at the moment of HCV seroconversion, while one started using PrEP after HCV seroconversion. All incident HCV infections were found between 2014 and 2018, and none of the participants with an incident HCV infection acquired HIV after HCV seroconversion. Three participants had borderline positive anti-HCV antibody signals at consecutive ACS study visits during follow-up and were not considered as incident HCV infections (range signal-to-cutoff between 1.10 and 3.60).

### Determinants associated with incident hepatitis C virus infection

Table 3 shows the characteristics at the last follow-up visit for those without incident HCV infection (*n* = 888), and at the cohort visit closest to the date of estimated incident HCV infection with available data for those with an incident HCV infection (*n* = 3). Incident HCV infection was more common among participants who reported receptive CAS (posterior HR = 1.43, 95% CrI = 0.45–4.74), 1–10 sex partners versus none (posterior HR = 2.07, 95% CrI = 0.62–7.46), recent IDU (posterior HR = 1.89, 95% CrI = 0.47–7.41), ever IDU (posterior HR = 1.74, 95% CrI = 0.47–6.68) and engaging in fisting (posterior HR = 1.71, 95% CrI = 0.45–6.16). All 95% CrIs of the posterior HR included one, suggesting a large degree of uncertainty.

**Table 3.** Characteristics and determinants associated with hepatitis C virus at end of follow-up or at visit of hepatitis C virus diagnosis or visit closest to incident hepatitis C virus infection, and prior and posterior estimates of determinants for hepatitis C virus infection among men who have sex with men without HIV participating in the Amsterdam Cohort Studies between 2012 and 2021, the Netherlands

	Not infected (*N* = 888), number (%) or median (IQR)	Infected (*N* = 3), number (%) or median (IQR)	*N* ^a^	Prior HR (95% CrI)	Posterior HR (95% CrI)
Age (years)^b,c^	43 (34–51)	44 (41–61)	3	1.00 (0.25–4.00)	1.38 (0.63–3.03)
Age, categories					
≤34 years	235 (26.5)	0 (0.0)	0	Reference	Reference
35–44 years	247 (27.8)	2 (66.7)	2	1.00 (0.25–4.00)	1.41 (0.41–4.93)
≥45 years	406 (45.7)	1 (33.3)	1	1.00 (0.25–4.00)	1.00 (0.29–3.30)
Country of birth					
The Netherlands	742 (83.6)	2 (66.7)	2	Reference	Reference
Other	146 (16.4)	1 (33.3)	1	1.00 (0.25–4.00)	1.23 (0.35–4.16)
Educational level					
Below college degree	194 (21.9)	1 (33.3)	1	Reference	Reference
College degree or higher	694 (78.1)	3 (66.7)	2	1.00 (0.25–4.00)	0.87 (0.27–3.14)
Receptive CAS	473 (53.3)	1 (33.3)	3	2.00 (0.50–8.00)	1.43 (0.45–4.74)
Number of sex partners					
None	55 (6.2)	0 (0.0)	0	Reference	Reference
1–10	662 (74.5)	3 (100.0)	3	1.50 (0.38–6.00)	2.07 (0.62–7.46)
≥11	171 (19.3)	0 (0.0)	0	1.50 (0.38–6.00)	1.13 (0.30–3.92)
IDU recent	7 (0.8)	0 (0.0)	3	2.00 (0.50–8.00)	1.89 (0.47–7.41)
IDU ever^d^	30 (3.4)	0 (0.0)	3	2.00 (0.50–8.00)	1.74 (0.47–6.68)
Chemsex^e^	377 (42.5)	0 (0.0)	3	1.50 (0.38–6.00)	0.87 (0.26–2.86)
Any SDU^f^	404 (45.4)	0 (0.0)	3	1.50 (0.38–6.00)	0.87 (0.25–2.73)
Engaging in group sex	213 (24.0)	0 (0.0)	3	1.50 (0.38–6.00)	0.94 (0.28–3.18)
Any STI	61 (6.9)	0 (0.0)	3	1.50 (0.38–6.00)	1.26 (0.34–4.71)
Engaging in fisting	83 (9.4)	0 (0.0)	3	2.00 (0.50–8.00)	1.71 (0.45–6.16)
Use of anal toys	98 (11.0)	0 (0.0)	3	1.50 (0.38–6.00)	1.29 (0.33–4.91)
Sharing of anal toys	30 (3.4)	0 (0.0)	3	1.50 (0.38–6.00)	1.41 (0.34–5.47)

CAS, condomless anal sex; CrI, credible interval; HCV, hepatitis C virus; HR, hazard ratio; IDU, injecting drug use; IQR, interquartile range; SDU, sexualized drug use; STI, sexually transmitted infection.

a Number of incident HCV infections with a given, time-updated determinant (three incident HCV infection in total).

b Per 10 years.

c Five participants with missing data on age.

d During the study period 2012–2021.

e Defined as the self-reported recent sexualized use of methamphetamine, γ-hydroxybutyric acid/γ-butyrolactone, mephedrone, ketamine, amphetamine, or ecstasy.

f Defined as any sexualized drug use, excluding alcohol, nitrates (poppers), erection stimulants, and cannabis.

### Phylogenetic analysis

Both individuals with an HCV-RNA positive test result were infected with subtype 1a. Phylogenetic analysis revealed that one HCV-RNA sequence, which was first detected in 2014, was closely related to sequences from MSM with HIV (Supplementary Figure S1, Supplemental digital content 1, https://links.lww.com/EJGH/B175). This HCV cluster was introduced in the early 2000s and seemed to be active for about 20 years without new infections of this strain detected since 2018. This cluster did not contain sequences from MSM using PrEP. HCV-RNA extracted from the other infection contained not enough HCV-RNA copies to perform full-length sequencing.

## Discussion

Among MSM without HIV enrolled in the ACS between 2012 and 2021, we found a low past/current prevalence of HCV at baseline and a low incidence of primary HCV infection during follow-up. Incident HCV infection was more common among individuals reporting receptive CAS, higher number of sexual partners, having recent and ever IDU, and engaging in fisting. None of the participants with a prevalent or incident HCV infection were using PrEP at the time of HCV seroconversion or acquired HIV after HCV seroconversion. Although phylogenetic analysis could only be performed on HCV-RNA from one MSM, this sequence appeared to intersperse with HCV-RNA sequences from MSM with HIV, suggesting potential overlap in transmission networks of MSM with and without HIV.

Historically, HCV infections among MSM in the Netherlands occurred predominately in MSM with HIV, and in the advent of universal DAA access in 2015, the incidence of HCV dramatically decreased in this key population [1]. Around the same time, PrEP became available for MSM without HIV in research settings, and HCV prevalence and incidence were found to be much higher than expected based on previous findings [13,17]. HCV transmission is known to occur within shared sexual networks of MSM with and without HIV using PrEP [14,15,17], and it could be conceived that these networks further extend to MSM without HIV who are not using PrEP. Indeed, a meta-analysis conducted in 2021 estimated a prevalence of 0.6% among MSM without HIV in the European region [12]. In our study, we found an even lower prevalence of 0.2% at baseline. Nevertheless, these results need to be considered in light of a previous study in the ACS, where no HCV infections were found among MSM without HIV between 1984 and 2012 [16].

The pooled incidence rate of HCV was 0.01 per 100 person-years among MSM without HIV not using PrEP and 0.8 per 100 person-years in MSM without HIV using PrEP (including studies with follow-up up to early 2018) [12,32]. Since then, much has changed in terms of epidemiological context, including the introduction of universal DAA access, widespread PrEP uptake, and shifts in sexual behavior patterns. While the observed incidence rate of 0.05 per 100 person-years is in line with these estimates, it fell somewhat on the lower bound of the reported range. The differences in reported incidence rates are likely due to variations in local epidemiology between countries, sexual behavior, calendar time, and health care coverages and protocols. In our study, we observed that both sexual (i.e. receptive CAS, higher number of sexual partners, and engaging in fisting) and drug use variables were associated with incident HCV infection. Although the CrI of the determinants with the strongest association with incident HCV infection included one, and thus have a high degree of uncertainty, these determinants were in line with those found among MSM with HIV and MSM using PrEP [17,31,33]. Considering the high degree of uncertainty of these determinants coupled with low incidence of HCV infection among MSM without HIV not using PrEP, behavioral interventions on these factors would probably bear limited benefits in reducing HCV incidence for MSM without HIV not using PrEP.

The WHO has recommended systematic screening for anti-HCV antibodies in what they term ‘high risk’ populations [34]. MSM are included in this list of populations, particularly if they have HIV or engage in sexual practices associated with HCV. Yet in these same guidelines, general screening is recommended if the prevalence of anti-HCV antibody positive serology is 2% or higher [34]. Given that the prevalence of HCV infection at baseline was much lower than 2% and the incidence of HCV infections was also low, systematic anti-HCV antibody screening would seem largely unnecessary in this population. Taken together, since the decline in HCV incidence after DAAs became available, we are in a phase of the HCV epidemic where case finding is becoming more important [1]. Perhaps HCV screening in MSM without HIV would be more efficient if based on an assessment of susceptible behaviors rather than systematically performed.

Our study has some limitations. First, the small number of incident HCV infections observed precluded the ability to perform multivariable analysis; however, given the infrequency of infections and similarity in determinants associated with HCV found compared with larger studies among MSM, multivariable analysis would unlikely lead to any novel findings. Second, data on some of the determinants known to be associated with HCV have not been assessed. For instance, no information was available on sharing straws when nasally administered drugs were used and the cohort lacked data on whether gloves were used or shared when engaging in fisting, and whether anal sex toys were cleaned between sex acts with multiple sexual partners [9,35]. Third, HCV-RNA testing results, treatment, and treatment outcomes were lacking for some of the individuals with a prevalent or incident HCV infection. This limited the number of HCV strains to phylogenetically compare with strains from MSM with and without HIV, and our ability to assess the potential for onward transmission among anti-HCV antibody positive cases. Fourth, some samples had been stored for several years. Prolonged storage, particularly with repeated freeze-thaw cycles, may have resulted in degraded samples, potentially affecting the RNA amplification needed for genotypic analysis. Fifth, the generalizability of our findings may be limited in settings where the epidemiology of HCV is different or where access to DAAs or PrEP remains restricted. Finally, the use of anti-HCV antibodies as a marker in our analysis did not allow for the identification of HCV reinfections. This limitation may have contributed to an underestimation of the true incidence rate as the HCV reinfection rate remains high in the MSM population [1,17].

In conclusion, few HCV infections were observed in a convenience sample of MSM without HIV residing in Amsterdam, the Netherlands. Behaviors associated with HCV acquisition in this study population were comparable to those found in previous studies among MSM. Phylogenetic analysis suggests the presence of shared sexual networks of MSM with HIV and MSM without HIV not using PrEP. In a setting with broad access to DAAs and PrEP, the low HCV incidence among MSM without HIV not using PrEP highlights that systematic screening of all MSM without HIV may be inefficient in terms of the number of tests per case, but rather screening based on specific sexual behaviors should be considered.

## Acknowledgements

The authors gratefully acknowledge the Amsterdam Cohort Studies (ACS) on HIV infection and AIDS, which is a collaboration between the Public Health Service of Amsterdam, Amsterdam UMC, Sanquin Blood Supply Foundation, Medical Center Jan van Goyen, and the HIV Focus Center of the DC‐Clinics. The authors wish to thank all ACS participants for their contribution, as well as the ACS study nurses, data managers, and laboratory technicians.

The Amsterdam Cohort Studies is part of the Netherlands HIV Monitoring Foundation and financially supported by the Center for Infectious Disease Control of the Netherlands National Institute for Public Health and the Environment, Bilthoven, the Netherlands. This work was supported by the STI Research and Development grant from the Public Health Service of Amsterdam, the Netherlands; and the Netherlands Organization for Health Research and Development (ZonMw; grant number 522004006).

K.H. and J.Kl. contributed to the conception and design of the work, performed data management, data analysis, and drafted the manuscript. A.B. contributed to the analysis of data and the interpretation of the data. M.B., L.v.d.H., J.K p., S.R., and J.S. contributed to the analysis of stored samples. A.M. and U.D. contributed to the design of the work. M.P. contributed to the conception and design of the work and the interpretation of data. All authors critically revised the manuscript, and all approved the final version.

### Conflicts of interest

A.B. has received speaker’s fees from Gilead Sciences, independent from the submitted work. L.v.d.H. has received project fees from MSD Animal Health, outside of the submitted work. M.P.’s institution has received speaker’s fees and independent scientific support from Gilead Sciences, Roche, MSD, and AbbVie, outside the submitted work. All other authors report no potential conflicts.

## Supplementary Material


