# Supplementary material for: Incidence of hepatitis C virus infection and associated determinants among men who have sex with men without HIV in Amsterdam, the Netherlands, between 2012 and 2021
Source: Eur J Gastroenterol Hepatol. 2025 Jun 23;37(10):1173–9. doi: 10.1097/MEG.0000000000003008 (PMC12382747; doi:10.1097/MEG.0000000000003008)
Supplement: Supplementary file 1 [file ejgh-37-1173-s001.docx]

**Supplementary Materials**

**Incidence of hepatitis C virus and associated determinants among MSM without HIV in Amsterdam, the Netherland, between 2012-2021**

Kris Hage, Jeffrey Koole, Anders Boyd, Amy Matser, Udi Davidovich, Margreet Bakker, Lia van der Hoek, Jelle Koopsen, Sjoerd Rebers, Janke Schinkel, Maria Prins

**Contents**

[**Supplementary Table 1.** Prior distributions and assigned priors in Bayesian analysis 2](#_Toc197330578)

[**Supplementary Table 2.** Description of the number of missing values for imputed variables 3](#_Toc197330579)

[**Supplementary Table 3.** Comparison of characteristics of MSM without HIV participating in the Amsterdam Cohort Studies (ACS) at baseline who are included vs. excluded in the incidence estimates, Amsterdam, the Netherlands, 2012-2021 4](#_Toc197330580)

[**Supplementary Figure 1.** Maximum-likelihood tree comparing HCV sequence from one MSM without HIV who had incident infection during follow-up visits in the Amsterdam Cohort Study between 2012-2021 (indicated by red star) with HCV sequences obtained from individuals with HIV in the Netherlands and other countries. 5](#_Toc197330581)

# **Supplementary Table 1.** Prior distributions and assigned priors in Bayesian analysis

| **Determinant** | **Assigned prior** | **Dichotomous determinants**  **HR (95% CrI)** | **Continuous determinants**  **HR (95% CrI)** |
| --- | --- | --- | --- |
| Older age | Unknown direction | 1.00 (0.25-4.00) | 1.00 (0.25-4.00) |
| College degree or higher | Unknown direction* | 1.00 (0.25-4.00) | 1.00 (0.25-4.00) |
| Born in a country other than the Netherlands | Unknown direction* | 1.00 (0.25-4.00) | 1.00 (0.25-4.00) |
| Receptive CAS | Probably harmful | 2.00 (0.50-8.00) | 1.25 (0.31-5.00) |
| IDU | Probably harmful | 2.00 (0.50-8.00) | 1.25 (0.31-5.00) |
| Ever IDU | Probably harmful | 2.00 (0.50-8.00) | 1.25 (0.31-5.00) |
| Chemsex | Possibly harmful | 1.50 (0.38-6.00) | 1.10 (0.28-4.40) |
| Any SDU | Possibly harmful | 1.50 (0.38-6.00) | 1.10 (0.28-4.40) |
| Group sex | Possibly harmful | 1.50 (0.38-6.00) | 1.10 (0.28-4.40) |
| Any STI | Possibly harmful | 1.50 (0.38-6.00) | 1.10 (0.28-4.40) |
| Higher number of sexual partners | Possibly harmful | 1.50 (0.38-6.00) | 1.10 (0.28-4.40) |
| Fisting | Probably harmful | 2.00 (0.50-8.00) | 1.25 (0.31-5.00) |
| Use of sex toys | Possibly harmful | 1.50 (0.38-6.00) | 1.10 (0.28-4.40) |
| Sharing of sex toys | Possibly harmful | 1.50 (0.38-6.00) | 1.10 (0.28-4.40) |

All priors are based on log-normal distribution with mean ln(HR) and variance 1/2.

Informative priors were assigned on the basis of determinants identified from previous studies for acute HCV infection among MSM with HIV and MSM using HIV PrEP. An asterisk indicates that for this determinant, no previous studies were available, and these priors were postulated.

Abbreviations: CAS, Condomless anal sex; CrI, Credible interval; HR, Hazard-ratio; IDU, Injecting drug use; MSM, Men who have sex with men; PrEP, Pre-exposure prophylaxis; SDU, Sexualized drug use; STI, Sexually transmitted infection

# **Supplementary Table 2.** Description of the number of missing values for imputed variables

| **Imputed variable** | **Number of missing observations (%)^†^** |
| --- | --- |
| Age | 20 (0.2) |
| Country of birth | 17 (0.1) |
| Educational level | 18 (0.2) |
| Receptive CAS | 2,682 (22.0) |
| Number of sexual partners | 2,643 (21.7) |
| IDU | 2,663 (21.9) |
| Ever IDU^‡^ | 20 (0.2) |
| Chemsex^§^ | 2,408 (19.8) |
| Any SDU^¶^ | 2,406 (19.8) |
| Engaging in group sex | 2,658 (21.8) |
| Any STI | 2,805 (23.0) |
| Engaging in fisting | 8,889 (73.0) |
| Use of anal toys | 8,890 (73.0) |
| Sharing of anal toys | 9,006 (74.0) |

**^†^** The denominator represents the total count of observations included in the longitudinal analyses, which is a total of 12,174 observations.

^‡^ During the study period 2012-2021.

^§^ Defined as the self-reported recent sexualized use of methamphetamine, γ-hydroxybutyric acid (GHB)/γ-butyrolactone (GBL), mephedrone, ketamine, amphetamine and/or XTC.

^¶^ Defined as any sexualized drug use, excluding alcohol, nitrates (poppers), erection stimulants and cannabis.

Abbreviations: CAS, Condomless anal sex; IDU, Injecting drug use; SDU, Sexualized drug use; STI, Sexually transmitted infection

| **Supplementary Table 3.** Comparison of characteristics of MSM without HIV participating in the Amsterdam Cohort Studies (ACS) at baseline who are included vs. excluded in the incidence estimates, Amsterdam, the Netherlands, 2012-2021 | | | | |
| --- | --- | --- | --- | --- |
|  | | **Included**  **(n=891)** | **Excluded**  **(n=33)** | ***p*** |
| Age, years**^†^** | | 36 (28-43) | 27 (22-37) | 0.04 |
|  | 18-34 | 380 (42.7) | 18 (54.6) | 0.05 |
|  | 35-44 | 328 (36.8) | 5 (15.2) |  |
|  | ≥45 | 178 (20.0) | 4 (12.1) |  |
| Born in the Netherlands^‡^ | | 740 (83.1) | 24 (72.7) | 0.75 |
| Educational level^§^ | |  |  | 0.58 |
|  | Less than college degree | 186 (20.9) | 8 (24.2) |  |
|  | College degree or higher | 704 (79.0) | 24 (72.7) |  |
| Residence in Amsterdam^¶^ | | 731 (82.0) | 20 (60.6) | 0.76 |
| Living situation^††^ | |  |  | 0.02 |
|  | Alone | 433 (48.6) | 21 (63.6) |  |
|  | With steady partner | 288 (32.3) | 3 (9.1) |  |
|  | With parents or caretakers | 29 (3.3) | 2 (6.1) |  |
|  | With others | 138 (15.5) | 6 (18.2) |  |
| Exclusively homosexual^‡‡^ | | 709 (79.6) | 25 (75.8) | 0.80 |
| PrEP naïve | | 876 (98.3) | 33 (100.0) | 1.00 |

Presented are *n* (%) or median (IQR).

**^†^** Missing data: n=11 of which n=5 and n=6 among those included and excluded, respectively

^‡^ Missing data: n=9 of which n=4 and n=5 among those included and excluded, respectively

^§^ Missing data: n=2 of which n=1 and n=1 among those included and excluded, respectively

^¶^ Missing data: n=12 of which n=4 and n=8 among those included and excluded, respectively

^††^ Missing data: n=4 of which n=3 and n=1 among those included and excluded, respectively

^‡‡^ Missing data: n=5 of which n=4 and n=1 among those included and excluded, respectively

Abbreviations: ACS, Amsterdam Cohort Studies; HIV, Human immunodeficiency virus; IDU, Injecting drug use; MSM, Men who have sex with men; PrEP, Pre-exposure prophylaxis

#


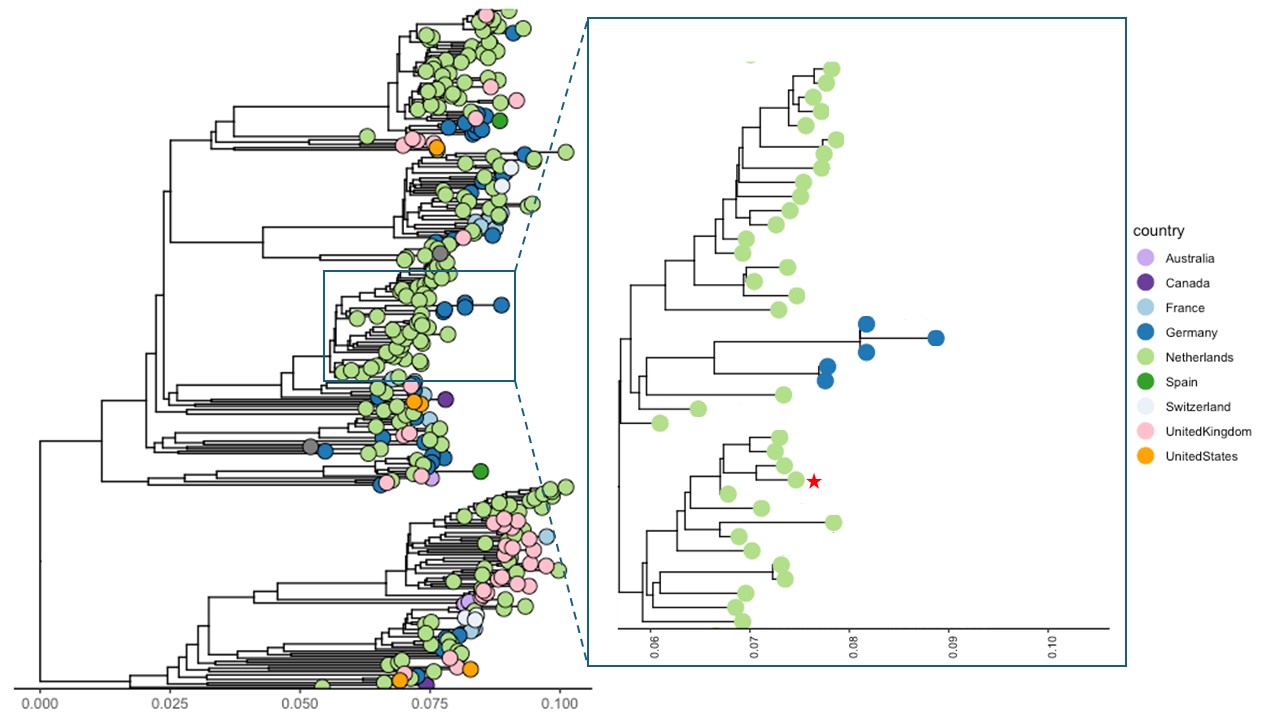


# **Supplementary Figure 1.** Maximum-likelihood tree comparing HCV sequence from one MSM without HIV who had incident infection during follow-up visits in the Amsterdam Cohort Study between 2012-2021 (indicated by red star) with HCV sequences obtained from individuals with HIV in the Netherlands and other countries.

Coloured tips denote country of sample collection. X-axis represents genetic distance.
